# Lipopolysaccharide Inhibits Autophagy and Promotes Inflammatory Responses via p38 MAPK-Induced Proteasomal Degradation of Atg13 in Hepatic Stellate Cells

**DOI:** 10.1155/2022/9603989

**Published:** 2022-09-13

**Authors:** Yuchao Wu, Yajuan He, Fei Wang, Naijuan Yao, Yingren Zhao, Zhen Tian

**Affiliations:** ^1^Department of Infectious Diseases, The First Affiliated Hospital of Xi'an Jiaotong University, Xi'an City, Shaanxi Province, China; ^2^Department of Ultrasound, The First Affiliated Hospital of Xi'an Jiaotong University, Xi'an City, Shaanxi Province, China

## Abstract

**Background:**

Inflammation plays a critical role in the progression of acute-on-chronic liver failure (ACLF). Atg13 is a vital regulatory component of the ULK1 complex, which plays an essential role in the initiation of autophagy. Previously, hepatic stellate cells (HSCs) were considered to be noninflammatory cells that contribute only to hepatic fibrosis. Recently, it has been found that HSCs can secrete inflammatory cytokines and participate in hepatic inflammation. Autophagy and proteasome-mediated degradation constitute two major means of protein turnover in cells. Autophagy has been shown to regulate inflammation, but it is unclear whether ubiquitin (Ub)-proteasome system (UPS) is involved in inflammatory responses in HSCs during ACLF.

**Methods:**

Clinical data were collected from ACLF patients, and surgically resected paraffin-embedded human ACLF liver tissue specimens were collected. The expression of Atg13 was assessed by quantitative real-time polymerase chain reaction (qRT-PCR) and Western blotting. Secretion of IL-1*β* was assessed by ELISA. Atg13 was knocked down by siRNA in LX2 cells. Coimmunoprecipitation assay was used to detect protein binding and polyubiquitination of Atg13. In vitro tests with LX2 cells were performed to explore the effects and regulation of p38 MAPK, Atg13, UPS, autophagy, and inflammation.

**Results:**

Serum lipopolysaccharide (LPS) was positively associated with disease severity in ACLF patients, and p38 MAPK was overexpressed in ACLF liver tissue. We evaluated the role of Atg13 in HSC inflammation and explored the possible underlying mechanisms. Inflammatory factors were upregulated via activation of p38 MAPK and inhibition of autophagy in LX-2 cells. Expression of Atg13 was decreased in LPS-incubated LX2 cells. Atg13 knockdown markedly inhibited autophagy and promoted LPS-induced inflammation in LX2 cells. Our in vitro experiments also showed that LPS induced depletion of Atg13 via UPS, and this process was dependent on p38 MAPK.

**Conclusions:**

LPS induces proteasomal degradation of Atg13 via p38 MAPK, thereby participating in the aggravation of LPS-induced autophagy inhibition and inflammatory responses in LX2 cells. Atg13 serves as a mediator between autophagy and proteasome. Modulation of Atg13 or proteasome activity might be a novel strategy for treating HSC inflammation.

## 1. Introduction

Liver failure, including acute, chronic, and acute-on-chronic liver failure, is a rare but dramatic clinical syndrome characterized by massive hepatocyte death and overactivation of hepatic inflammation [1]. Acute-on-chronic liver failure (ACLF), which is characterized by an acute deterioration of liver function in patients with preexisting chronic liver disease, usually results in hepatocellular dysfunction and has a high mortality rate [2]. Apart from liver transplantation, few effective therapies are available, and ACLF continues to be a large therapeutic challenge [3]. The precise molecular mechanisms for the pathogenesis of ACLF have not been clarified. Thus, exploring ACLF-associated molecules may enable the development of strategies to improve the prognosis for patients with ACLF.

Hepatic stellate cells (HSCs) are resident mesenchymal cells that account for 15% of total resident cells in the liver [4]. Although the role of HSC activation in liver fibrosis has been widely accepted, it is not fully understood whether and how HSCs participate in hepatic inflammation. HSCs are located in the liver sinusoids together with sinusoidal endothelial cells (SECs) and Kupffer cells. Considering their anatomical position, HSCs seem to respond to inflammatory stimuli from liver sinusoids. Recent studies have found that activated HSCs may release inflammatory cytokines, such as IL-1*β*, IL-6, and TNF-*α* [5]. HSCs from both humans and rodents release inflammatory cytokines, and these inflammatory cytokines then participate in the disease pathogenesis of hepatocellular carcinoma and immune-mediated hepatitis [6].

Bacterial lipopolysaccharide (LPS), a cell wall component of gram-negative bacteria, is among the strongest known inducers of inflammation [7]. A previous study found an elevated serum LPS level in patients with ACLF due to changes in gut permeability and increased bacterial translocation [8]. LPS is associated with hepatic fibrogenesis through direct interactions with HSCs [9]. However, it remains unknown how LPS regulates HSC inflammation.

Proteolysis in eukaryotic cells is mainly mediated by the ubiquitin (Ub)-proteasome system (UPS) and the autophagy-lysosome system (ALS). Proteasomal degradation typically allows selective turnover of short-lived proteins, whereas autophagy is often considered a process that favors degradation of long-lived proteins [10]. Autophagy and UPS are strongly interconnected, and deregulation of either process likely underpins many diseases linked to defective protein degradation such as Alzheimer's disease [11]. There is increasing evidence that autophagy limits NLRP3 inflammasome activation and participates in hepatic inflammation [12, 13]. However, no studies have examined whether UPS regulates inflammasomes and participates in hepatic inflammation.

Atg13 is a vital regulatory component of the Atg13-ULK1 complex, which plays an essential role in the initiation step of autophagy [14]. In response to LPS stimulation, the Atg13-ULK1 complex is disrupted; autophagy is inhibited through p38 MAPK; and autophagy inhibition is dispensable for inflammasome activation and IL-1*β* induction in HSCs [15]. Recent studies have shown that the protein stability of the Atg13-ULK1 complex is tightly controlled by the ubiquitin modification system to regulate autophagy in mammalian cells. Under prolonged nutrient starvation, ULK1 autophosphorylation promotes its interaction with Cullin/KELCH-LIKE PROTEIN20, a substrate adaptor of Cul3 ubiquitin ligase, for K48-linked ubiquitylation and degradation [16]. In contrast, under normal nutrient conditions, the RING-type E3 ligases SINAT1 and SINAT2 regulate the ubiquitylation and degradation of Atg13 [17], and LUBAC and OTULIN cooperatively regulate autophagy initiation and autophagosome maturation by mediating the linear ubiquitination and the stabilization of Atg13 [18]. However, it remains unclear whether the ubiquitination and the stabilization of Atg13 are involved in LPS-induced HSC inflammation.

The aim of the present study was to evaluate whether and how LPS is involved in HSC inflammation via Atg13.

## 2. Materials and Methods

### 2.1. Patients

From January 2018 to September 2019, a total of 56 patients diagnosed with ACLF were enrolled in our study at the First Affiliated Hospital of Xi'an Jiaotong University, Shaanxi, China. All participants provided written informed consent, and the study was approved by the Research Ethics Committee of the First Affiliated Hospital of Xi'an Jiaotong University (Ethic approval number: XJTUFH 2018 G-205). Blood samples were collected at admission and stored at –80°C within 2 h.

Patients were diagnosed with ACLF based on the criteria of Asian Pacific Association for the Study of the Liver (APASL) [19]: (1) serum bilirubin ≥ 85 mol/L; (2) INR ≥ 1.5 or prothrombin activity ≤ 40%; (3) any degree of encephalopathy and/or clinical ascites within 4 weeks; (4) and an evidence of ongoing chronic liver diseases. Patients who were diagnosed with ACLF and aged 18 to 75 years were included. We calculated the Model for End-Stage Liver Disease (MELD) score using the standard formula: 11.2^∗^ln (INR) + 9.57^∗^ln (creatinine, in mg per decilitre) + 3.78^∗^ln (bilirubin, in mg per decilitre), with a lower limit of 1 for all variables. During the same period, age- and sex-matched cirrhotic participants were recruited as controls.

### 2.2. Antibodies and Reagents

LPS (#L2880), SB230580 (#S8307), Anisomycin (#A5862), SP600125 (#S5567), and MG132 (#474790) were purchased from Sigma-Aldrich. LY3214996 (#HY100494), Bafilomycin A1 (#HY-100558), and Cycloheximide (#HY-12320) were purchased from MedChem Express. The following primary antibodies were used: anti-p38 MAPK (#9217, Cell Signaling), antiphospho-p38 MAPK (#9216, Cell Signaling), anti-NLRP3 (#15101, Cell Signaling), anti-IL1 (#12703, Cell Signaling), anti-Atg13 (#13273, Cell Signaling), anti-LC3 (#8899, Cell Signaling), anti-p62 (#5114 Cell Signaling), anti-ULK1 (#8054, Cell Signaling), anti-FIP200 (#12436, Cell Signaling), anti-ubiquitin (#134953, Abcam), anti-Atg5 (#9980, Cell Signaling), anti-Myc (#2276, Cell Signaling), anti-GST (#2625, Cell Signaling), and *β*-actin as a loading control (#4970, Cell Signaling).

### 2.3. Estimation of IL-1*β* and LPS

Secretion of IL-1*β* levels was detected by utilizing Human IL-1*β* ELISA Kit (SLB50, R&D Systems) according to the manufacturer's protocol. Samples and standards were run in duplicate.

Serum LPS levels were detected by utilizing Human LPS ELISA Kit (ML061109, Mlbio) according to the manufacturer's protocol. Samples and standards were run in duplicate.

### 2.4. Histological Sampling

We collected surgical resected paraffin-embedded human ACLF liver tissue specimens (5 cases) and cirrhotic liver tissue specimens (5 cases) from the Department of Pathology, the First Affiliated Hospital of Xi'an Jiaotong University, with the approval of the Institutional Review Board. Immunoreactions were performed on selected liver sections. Antigens were detected by one of the following primary antibodies, followed by appropriate secondary antibodies: anti-p38 (#8690, Cell Signaling Technology, Danvers, MA, USA). The slides were then observed under a Nikon Eclipse microscope (Tokyo, Japan) coupled to a digital camera.

### 2.5. Cell Culture

The human hepatic stellate cells LX2 cells, HEK-293, and HL-7702 cells were cultured in Dulbecco's modified Eagle's medium supplemented with 10% fetal bovine plasma and 2 mM L-glutamine at 37°C in a 95% air, 5% CO_2_-humidified atmosphere. The THP-1 cells were cultured in RPMI 1640 medium supplemented with 10% fetal bovine plasma and 2 mM L-glutamine at 37°C in a 95% air, 5% CO2-humidified atmosphere.

### 2.6. Plasmid Transfection

HEK-293 cells were transfected with plasmids by using Lipofectamine 2000 reagent (Invitrogen, 11668027). Whole-cell lysates were prepared after 48 h plasmid transfection.

### 2.7. Coimmunoprecipitation Assay for Protein Binding

Cell lysates were prepared in lysis buffer and incubated with Protein A/G-Sepharose beads at 4°C for 3 h. Precleared lysates were incubated with appropriate antibody at 4°C for 12 h with gentle rotation. Protein A/G-Sepharose beads were added and incubated for 3 h. Immunoprecipitants were collected by centrifugation, washed five times with lysis buffer, heated at 100°C for 5 min, and subjected to SDS-PAGE.

### 2.8. Quantitative Real-Time Polymerase Chain Reaction (qRT-PCR)

Total RNA was extracted from cultured cells using the TRIzol reagent (Thermo, Life Technologies, Carlsbad, CA, USA). Reverse transcription was performed using the RevertAid First Strand cDNA Synthesis Kit (Thermo Scientific, Rockford, AL, USA). The relative abundance of mRNA in each sample was determined by qRT-PCR using the SYBR Premix ExTaq™II kit (TaKaRa, Dalian, China) and specific primers (designed and synthesized by TaKaRa, listed below) on an iQTM multicolor real-time PCR detection system (Bio-Rad, Hercules, CA, USA). Data were analyzed using the ΔΔCT method, and *β*-actin served as the internal control. The results are presented as mean ± SD of triplicate reactions from three separate experiments.

### 2.9. Immunoblotting

Protein extracts were prepared from cells by RIPA lysis buffer supplemented with complete EDTA-free protease inhibitor cocktail tablets (Roche Applied Science, Basel, Switzerland) and phosphatase inhibitor cocktails (Sigma-Aldrich). Protein samples (50 *μ*g) were loaded onto SDS-PAGE gels and transferred onto PVDF membranes. After blocking in 5% evaporated milk at room temperature for 2 h, the membranes were then incubated with the indicated primary antibodies in 5% evaporated milk in TBS plus 0.1% Tween 20 overnight at 4°C. Signals were developed using a chemiluminescent substrate and visualized through X-ray films.

### 2.10. siRNA Transfection

LX2 cells (1.5 × 10^5^) were transfected with 100 nM nontargeting control and human Atg13 siRNA (Thermo Scientific, 122699) using Lipofectamine 2000 (Invitrogen, 11668027) according to the manufacturer's protocol. Twenty-four h posttransfection, cells were analyzed by immunoblotting.

### 2.11. Statistical Analysis

The results are expressed as the means ± standard deviation. Statistical analysis was performed using SPSS software 13.0 (SPSS, Inc., Chicago, IL, USA). The Shapiro-Wilk test and Levene statistic were used to evaluate the normality and homogeneity, respectively, of the variance. According to the situation, *t* tests or Mann–Whitney *U* tests were used to evaluate differences between two groups; correlations between two quantitative groups were analyzed with Pearson or Spearman correlation tests. The *χ*^2^ test was used for comparisons between two groups. The reported *P* values are two-sided, and *P* values < 0.05 were considered statistically significant.

## 3. Results

### 3.1. Serum LPS Level Increases in ACLF

**Figure 1:**
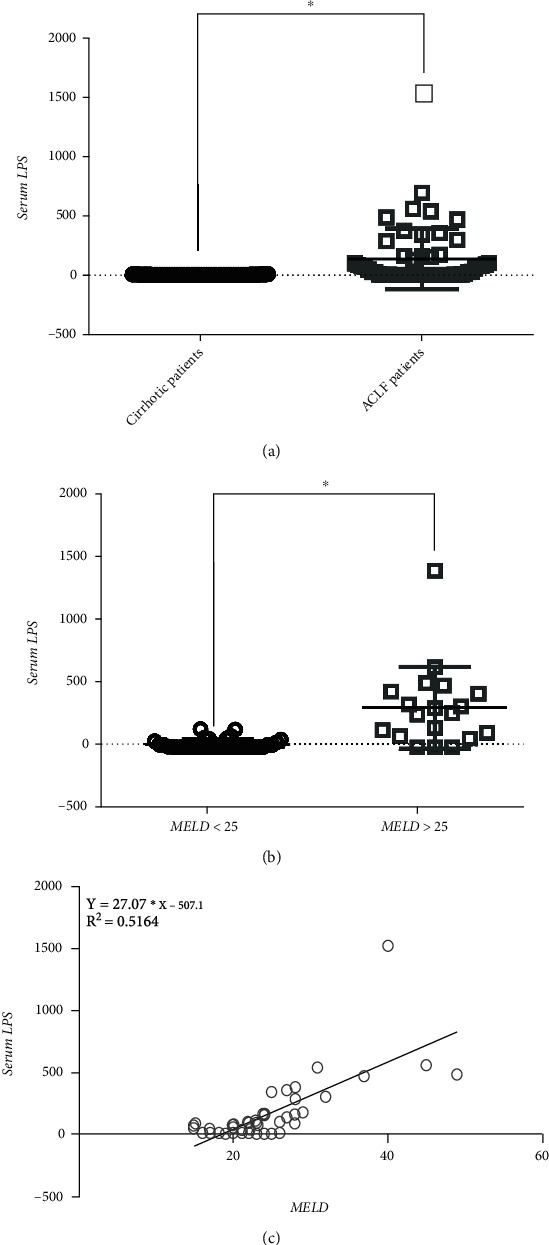
Increased serum LPS level is associated with poor prognosis of ACLF. Plasma from acute-on-chronic liver failure (ACLF) patients at admission and from cirrhotic patients were tested for LPS. (a) LPS level in ACLF patients was significantly increased compared to cirrhotic controls. (b) ACLF patients with MELD score > 25 displayed higher LPS level compared to patients with MELD score ≤ 25; (c) Positive correlation between serum LPS level and MELD score. ^∗^*P* < 0.01.

Liver failure is an inflammation-mediated hepatocellular injury process, and LPS levels in serum are elevated in patients with acute liver failure (ALF) and ACLF due to increased gut permeability. Here, we investigated serum LPS levels in patients with ACLF (Table 1). We found a significantly higher serum LPS concentration in patients with ACLF than in those with cirrhosis (177.5 ± 64.52 vs. 5.830 ± 0.3019, *P* < 0.05) (Figure 1(a)). Previously, we found that MELD score > 25 was associated with short-term mortality in ALF patients [20]. We also found that ACLF patients with MELD score > 25 had significantly greater mortality [21]. We then divided these ACLF patients into a low-risk group (MELD score ≤ 25) and a high-risk group (MELD score > 25). Patients in the high-risk group had higher LPS levels than patients in the low-risk group (217.0 ± 70.56 vs. 83.68 ± 31.79, *P* < 0.05) (Figure 1(b)). Furthermore, a positive correlation was observed between LPS level and MELD score (*R*^2^ = 0.5164, *P* < 0.01) (Figure 1(c)).

### 3.2. Expression of p38 MAPK in ACLF

**Figure 2:**
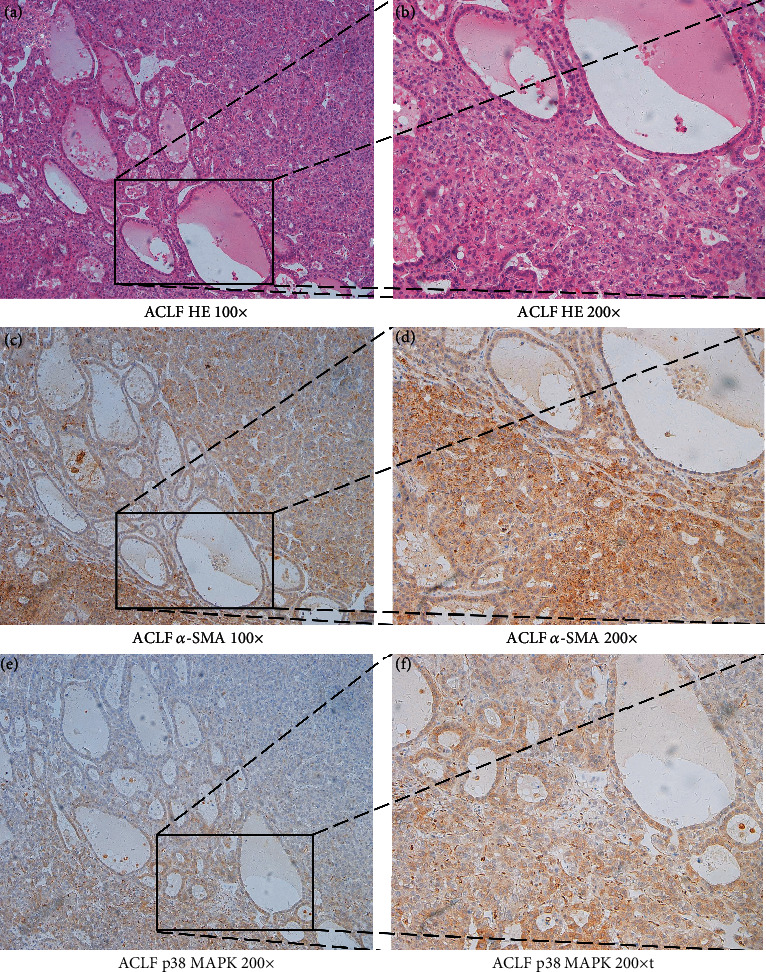
Immunohistochemical staining were conducted in liver tissues from patients with ACLF. The tissues from ACLF patients were firstly stained with hematoxylin-eosin (HE) (a, b), then the expression of *α*-SMA (c, d) and p38 MAPK (e, f). Original magnification 100x (a, c, and e) or 200x (b, d, and f).

It has been demonstrated that phosphorylation levels of p38 MAPK are significantly increased in HSCs treated with LPS, leading to HSC activation and IL6 secretion [22]. LPS is known to engage Toll-like receptor 4 (TLR4) and p38 MAPK in inducing inflammatory response. To examine whether this pathway participates in the pathogenesis of ACLF, we tested in vivo expression of p38 MAPK in liver tissues from ACLF patients. The specimens of patients with ACLF displayed positive immunoreactivity for p38 MAPK, and *α*-SMA was also expressed (Figures 2(a)−2(f)). As *α*-SMA is a marker of HSCs, we assumed that p38 MAPK may participate in the progression of HSC inflammation.

### 3.3. LPS Promotes Inflammation in HSCs through p38 MAPK

**Figure 3:**
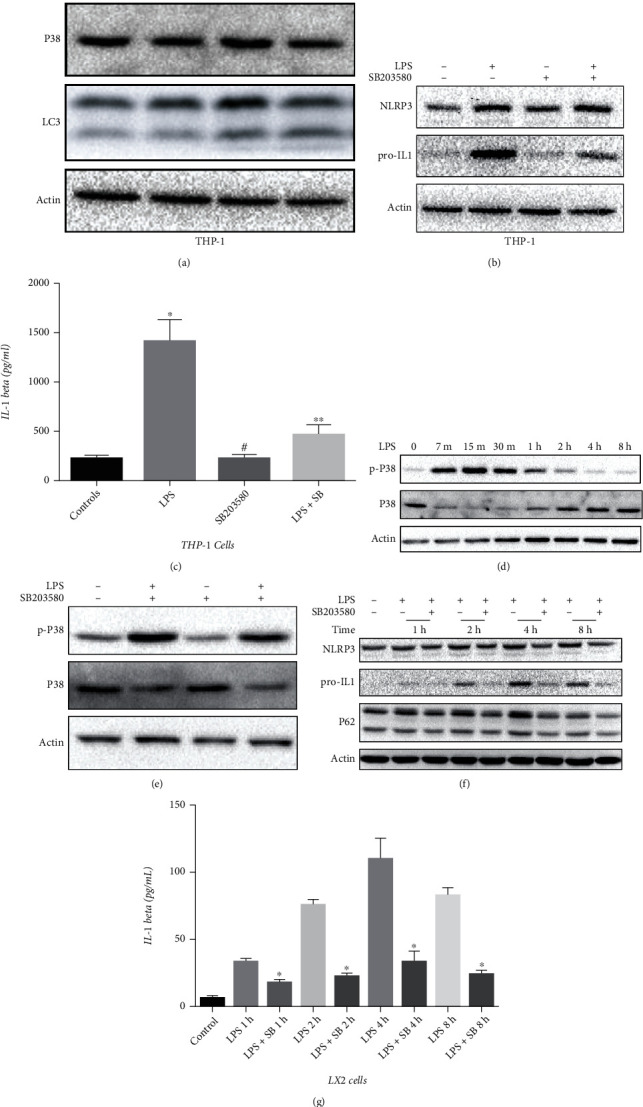
LPS promotes inflammation via p38 MAPK in HSCs. (a–c) THP-1 cells in the logarithmic growth phase were treated with 1 *μ*g/mL LPS for 15 min for the detection of p38 MAPK or for 4 h for the detection of inflammation. (d) LX-2 cells in the logarithmic growth phase were treated with 1 *μ*g/mL LPS in a time-dependent manner (0-8 h). (e–g) LX-2 cells in the logarithmic growth phase were treated with or without 20 *μ*M SB203580 for 30 min, then cells were treated with 1 *μ*g/mL LPS for 15 min for the detection of p38 MAPK or in a time-dependent manner (0-8 h) for the detection of inflammation.

LPS is known to an engage inflammatory response in THP-1 cells. We showed here that SB203580, a chemical inhibitor of p38 MAPK, effectively suppressed LPS-induced phosphorylation of p38 MAPK and NLRP3 inflammasome activation in THP-1 cells (Figures 3(a)−3(c)). In the present study, LPS induced time-dependent activation of p38 MAPK, and SB203580 suppressed this phosphorylation (Figures 3(d) and 3(e)) in LX2 cells. We also found that LPS induced a time-dependent activation of the inflammatory response in LX2 cells, and the use of SB203580 significantly attenuated LPS-induced NLRP3 activation and production of IL-1*β* (Figures 3(f) and 3(g)). In addition, we found a reversal of LPS-induced accumulation of p62, which means autophagy inhibition, in LX2 cells, cotreated with SB203580 (Figure 3(f)).

### 3.4. LPS Inhibits Autophagy in HSCs through p38 MAPK

**Figure 4:**
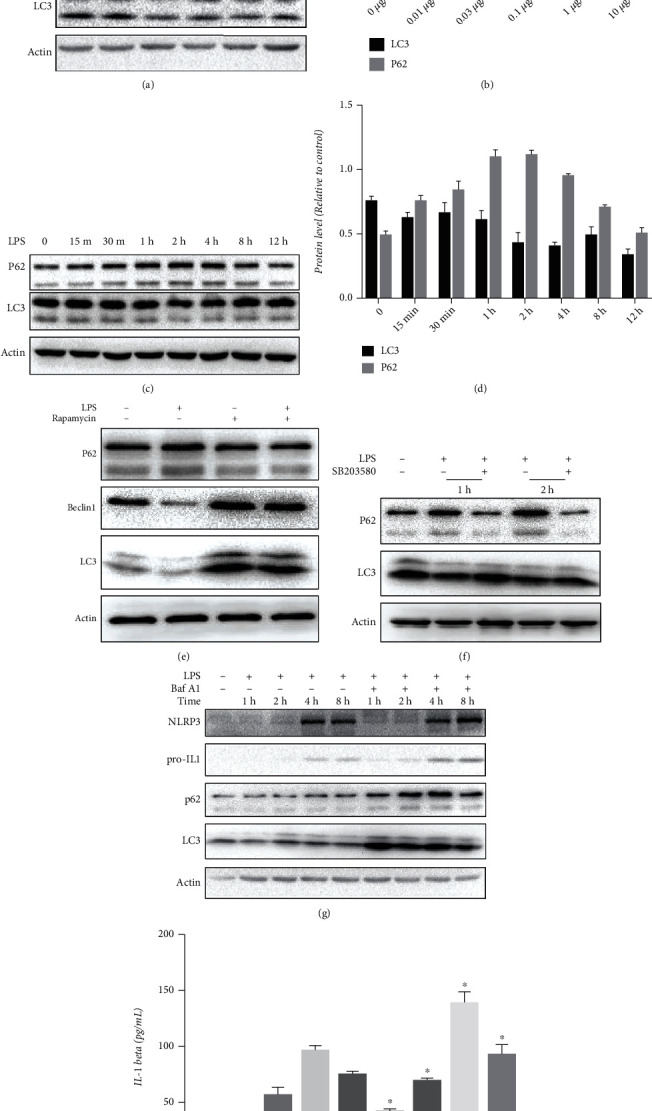
LPS inhibits autophagy via p38 MAPK in HSCs. (a, b) LX-2 cells in the logarithmic growth phase were treated with different concentrations of LPS (0-10 *μ*g/mL) for 4 h. (c, d) LX-2 cells in the logarithmic growth phase were treated with 1 *μ*g/mL LPS in a time-dependent manner (0-12 h). (e, f) LX-2 cells in the logarithmic growth phase were treated with 1 *μ*g/mL LPS together with or without 50 nM rapamycin or 20 *μ*M SB203580 for 4 h for the detection of autophagy. (g, h) LX-2 cells in the logarithmic growth phase were treated with 1 *μ*g/mL LPS together with or without 200 nM Bafilomycin A in a time-dependent manner (0-8 h) for the detection of autophagy.

To understand the control and release of autophagic suppression of inflammation, we first tested whether LPS modulates autophagy in HSCs. We treated LX2 cells with LPS and found a dose-dependent decrease in the level of autophagy marker LC3-II, the lipidated form of microtubule-associated protein 1A/1B-light chain 3 (LC3), and an increase in the level of autophagy adapter protein p62 (Figures 4(a) and 4(b)). Next, we evaluated the time course of autophagy in response to LPS in LX2 cells. When LX2 cells were treated with 1 *μ*g/mL of LPS, LC3-II expression decreased, whereas p62 expression first increased and then decreased after 4 h (Figures 4(c) and 4(d)).

To further confirm that LPS inhibits autophagy in LX2 cells, we used rapamycin, an initiator of autophagy, and Bafilomycin A1 (Baf A1), which is known to block the fusion of autophagosomes with lysosomes, together with LPS. Rapamycin inhibited LPS-induced changes in LC3-II and p62 (Figure 4(e)). Exposure to Baf A1 significantly promoted LPS-induced accumulation of p62, NLRP3 activation, and production of IL-1*β* (Figures 4(g) and 4(h)). Furthermore, SB203580 successfully alleviated LPS-induced inhibition of autophagy (Figure 4(f)).

### 3.5. LPS Promotes Depletion of Atg13 and Inflammation in LX2 Cells

**Figure 5:**
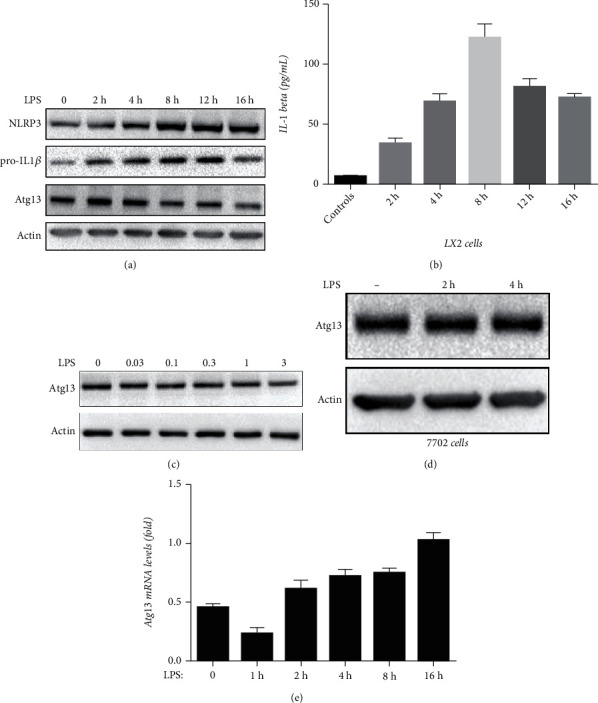
LPS promotes depletion of Atg13 and inflammatory responses in LX2 cells. LPS (1 *μ*g/mL, 0-16 h) suppressed expression of Atg13 in a time-dependent manner. (c) LPS (0-3 *μ*g/mL, 4 h) suppressed expression of Atg13 in a dose-dependent manner. (a, b) LPS (1 *μ*g/mL, 0-16 h) activated NLRP3 inflammasome activation and IL-1*β* secretion in a time-dependent manner. (d) LPS (1 *μ*g/mL, 0-4 h) did not alter the expression of Atg13 in HL-7702 cells. (e) LPS (1 *μ*g/mL, 0-16) did not alter the mRNA levels of Atg13 in LX2 cells.

We treated LX2 cells with 1 *μ*g/mL LPS for different time periods and found that the expression of Atg13 decreased in a time-dependent manner after incubation with LPS (Figure 5(a)). We next treated LX2 cells with different doses of LPS for 8 h; as shown in Figure 5(c), the expression of Atg13 decreased in a dose-dependent manner. Moreover, we found that the use of LPS led to the activation of inflammation in LX2 cells, given that the expression of NLRP3 and pro-IL1*β* and the secretion of IL-1*β* increased (Figures 5(a) and 5(b)). Interestingly, the effect of LPS on Atg13 appeared to be specific for HSCs since LPS did not alter the expression of Atg13 in liver parenchyma cells, HL-7702 cells (Figure 5(d)).

To further determine the time at which LPS downregulates Atg13 expression, the mRNA levels of Atg13 were measured by qRT-PCR, the primers used in qRT-PCR are shown in (Table 2. As shown in Figure 5(e), Atg13 mRNA expression was mildly decreased after 1 h of LPS treatment; however, it returned to normal levels after 4 h of treatment and remained unchanged thereafter. These results suggested that the LPS-mediated Atg13 suppression was unrelated to Atg13 mRNA levels.

### 3.6. Decrease in Atg13 Inhibits Autophagy and Promotes LPS-Induced Inflammation

**Figure 6:**
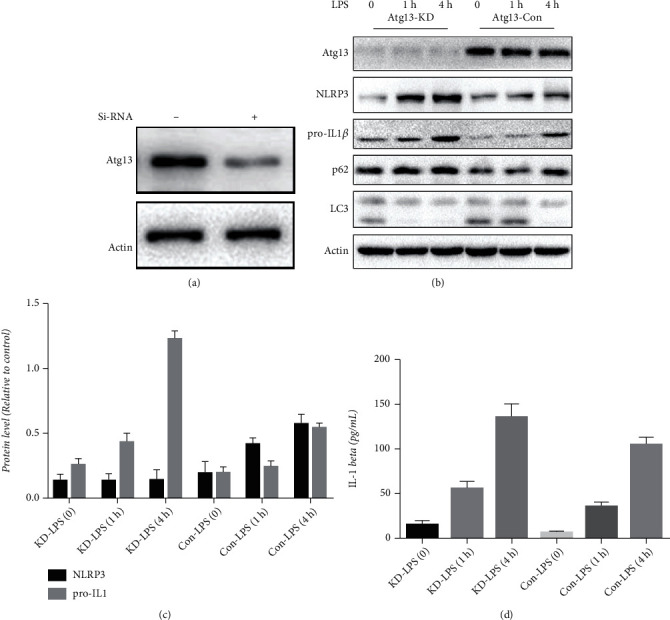
Decrease of Atg13 inhibits autophagy and promotes LPS-induced inflammatory responses in LX2 cells. (a) Atg13 siRNA successfully decreased expression of Atg13. (b–d) Decrease of Atg13 inhibited autophagy and promoted LPS (1 *μ*g/mL, 0-4 h)-induced NLRP3 inflammasome activation and IL-1*β* secretion.

To investigate whether the decrease of Atg13 in LX2 cells affects autophagy and inflammation induced by LPS, we used siRNA to knock down Atg13 in LX2 cells. Results of Western blotting confirmed the decreased expression of Atg13 (Figure 6(a)). We then treated wide-type and Atg13-knockdown LX2 cells with LPS. We found that the decrease in Atg13 promoted the inhibition of autophagy and inflammatory responses induced by LPS, as the expression of LC3 decreased, and p62, NLRP3, pro-IL1*β*, and secretion of IL-1*β* significantly increased (Figures 6(b)−6(d)).

### 3.7. LPS Promotes Depletion of Atg13 and Inflammation via p38 MAPK

**Figure 7:**
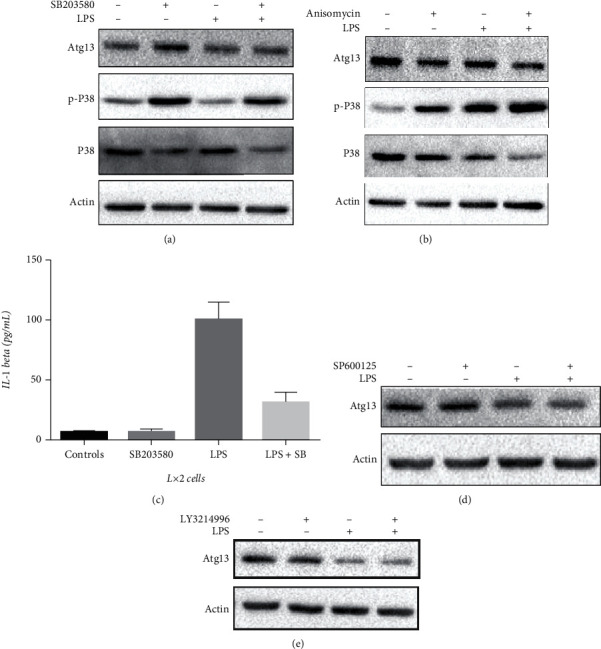
LPS promotes inflammation and induces Atg13 loss via p38 MAPK. (a, b) SB203580 (5 *μ*M) alleviated while Anisomycin (10 *μ*M) promoted LPS (1 *μ*g/mL, 4 h)-induced decrease of Atg13 (e, f). (c) SB203580 (5 *μ*M) alleviated LPS (1 *μ*g/mL, 4 h)-induced IL-1*β* secretion. (d, e) Neither SP600125 (40 *μ*M) nor LY3214996 (5 nM) altered expression of Atg13.

LPS is known to activate p38 MAPK in inducing the inflammatory response in THP-1 cells [23], as shown in Figures 3(a)−3(c). We showed here that SB203580 effectively suppressed LPS-induced depletion of Atg13, while the small molecule activator of p38 MAPK, Anisomycin, promoted depletion of Atg13 (Figures 7(a) and 7(b)). SB203580 inhibited LPS-induced inflammatory responses (Figure 7(c)). LPS has also been shown to regulate cellular functions via phosphorylating the MAPK superfamily, including ERK1,2 and JNK1,2 [24, 25]. Here, we found that the use of LY3214996, a JNK inhibitor, and SP600125, an ERK inhibitor, had no effect on the protein expression of Atg13 in LX2 cells (Figures 7(d) and 7(e)).

### 3.8. LPS Induces Depletion of Atg13 via p38 MAPK through Proteasome-Dependent Degradation

**Figure 8:**
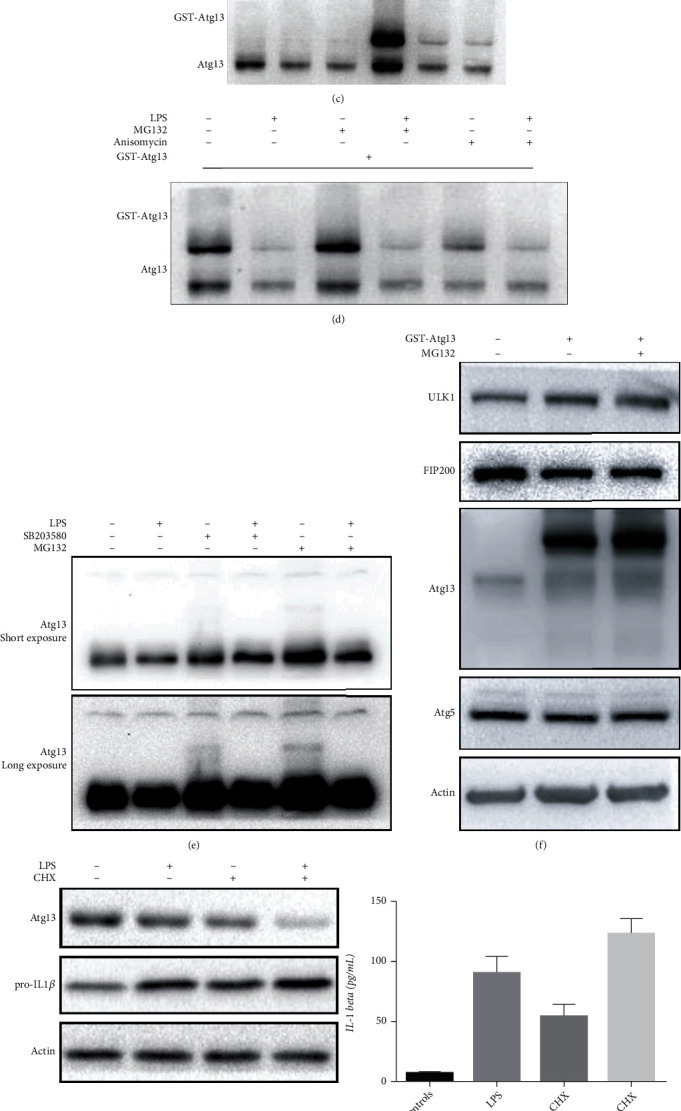
LPS induces Atg13 loss via proteasome-dependent degradation. (a) MG132 (0.5 *μ*M, 8 h) increased expression of Atg13, while Baf A1 (200 nM, 8 h) did not affect expression of Atg13 in LX2 cells. (b) HEK 293 cells expressing vector or GST-Atg13 and Myc-ubiquitin were treated with MG132 (0.5 *μ*M, 8 h) as indicated. Cell lysates were subject to Myc-tag or GST-tag affinity isolation and immunoblotted for Atg13 and ubiquitin expression. (c) LX2 cells expressing GST-Atg13 or not were treated with CHX (0-1 mg/mL, 2 h). (d) LX2 cells expressing GST-Atg13 were treated with LPS, MG132, or Anisomycin. (e) LX2 cells were treated with LPS, MG132, or Anisomycin as indicated; (f) LX2 cells expressing GST-Atg13 were treated with MG132; expression of ULK1, FIP200, and Atg5 was detected. (g, h) LX2 cells were treated with LPS, CHX, or MG132 as indicated; expression of pro-IL1*β* and secretion of IL-1*β* were detected.

Autophagy and proteasome-mediated degradation constitute the two major means of protein turnover in cells. Atg13 is an important component of the ULK1 complex, which plays an essential role in the initiation step of autophagy. We next determined whether autophagy or proteasome-mediated degradation contributed to the degradation of Atg13. We treated LX2 cells with MG132, the inhibitor of proteasome, and Baf A1, the inhibitor of autophagy. Our results showed that the use of MG132 significantly increased the expression of Atg13, while Baf A1 did not affect the expression of Atg13 (Figure 8(a)).

Polyubiquitination is the major means of targeting proteins for proteasome degradation. Therefore, we examined whether Atg13 is directly ubiquitinated. Empty vector or GST-tagged Atg13 together with Myc-tagged ubiquitin was expressed in HEK293 cells treated with or without MG132. It was immunoprecipitated with an anti-MYC antibody, and the precipitate was blotted with an anti-Atg13 antibody, or we performed the immunoprecipitation in reverse, showing that Atg13 was directly ubiquitinated (Figure 8(b)).

We then examined the protein stability of Atg13. Namely, LX2 cells were treated with cycloheximide (CHX), a protein synthesis inhibitor in the absence or presence of LPS. In CHX-treated cells, Atg13 protein was gradually reduced, and LPS significantly accelerated Atg13 reduction (Figure 8(g)). In a similar manner, ectopically expressed Atg13 was also gradually turned over (Figure 8(c)). Treatment of cells with the proteasome inhibitor MG132 increased endogenous and exogenous Atg13 (Figure 8(d)).

Moreover, we investigated whether LPS promotes Atg13 degradation via UPS. As shown in Figures 8(d)−8(f), treatment with SB203580 or MG132 reversed LPS-induced depletion of Atg13, and Anisomycin promoted the depletion of Atg13. We next transfected empty vector and GST-Atg13 to HEK293 cells, and we found that MG132 inhibited exogenous Atg13 degradation, while it did not affect the expression of ULK1 and FIP200, two essential components of the ULK1 complex and Atg5 (Figure 8(f)).

### 3.9. UPS Regulates LPS-Induced Depletion of Atg13 and Inflammation in LX2 Cells

We next examined whether the regulation of UPS affects inflammatory responses in LX2 cells. Our data revealed that CHX promoted LPS-induced depletion of Atg13 and inflammatory responses (Figure 8(g)), while MG132 alleviated LPS-induced depletion of Atg13 and inflammatory responses (Figure 8(h)).

## 4. Discussion

It has been known for years that autophagy negatively controls inflammasome activity and that a decrease in autophagic activity correlates positively with the inflammatory response [12]. ACLF is a life-threatening disease that is characterized by overactivation of hepatic inflammation [26]. Our data showed that serum LPS levels were significantly higher in patients with ACLF due to increased gut permeability; meanwhile, serum LPS correlated with MELD score and was associated with disease severity in patients with ACLF.

Activation of HSCs is the central step during liver fibrogenesis. Some recent studies have suggested that HSCs replay inflammation signaling from the sinusoid to parenchyma, given that HSCs from both humans and rodents produce inflammatory cytokines that promote hepatocellular carcinoma and immune-mediated hepatitis. However, few studies have focused on the underlying mechanism of HSC inflammation. Our recent work has shown that reactive oxygen species activate the NLRP3 inflammasome and promote inflammation in HSCs [20]. In the present study, we first confirmed that LPS could activate NLRP3 inflammasomes and promote inflammation. The stimulation by LPS inhibited autophagy via p38 MAPK, and this inhibition was necessary for LPS-induced inflammasome activation in LX2 cells. Our data showed that SB203580 successfully inhibited LPS-induced autophagy inhibition and inflammasome activation.

Autophagy is a conserved intracellular process by which cytoplasmic materials are delivered to lysosomes for degradation [27]. More than 40 autophagy-related proteins (ATGs) have been identified thus far. Atg13 is an essential factor required for autophagy regulation. Together with Atg101, FIP200, and ULK1, Atg13 constitutes the Atg13-ULK1 complex, which plays an essential role in the initiation step of autophagy: receiving signals of nutrient status, recruiting downstream autophagy-related proteins, and governing autophagosome formation. In the present study, incubation with LPS led to the decreased expression of Atg13 in HSCs. To investigate whether the decrease of Atg13 affected autophagy and LPS-induced HSC inflammation, we knocked down Atg13 in LX2 cells via siRNA and found that the decrease in Atg13 inhibited autophagy and promoted inflammation induced by LPS.

MAPKs, including p38, ERK, and c-JNK, are members of a ubiquitous protein serine/threonine kinase family responsible for signal transduction in eukaryotic organisms [28]. LPS is known to engage Toll-like receptor 4 (TLR4) and then activate p38 MAPK [29]. We showed here that LPS induced p38 MAPK phosphorylation in LX2 cells. SB203580, a specific inhibitor of p38 MAPK, successfully inhibited LPS-induced phosphorylation of p38 MAPK and secretion of IL-1*β*. Our data also revealed that SB203580 inhibited and Anisomycin promoted LPS-induced Atg13 loss in LX2 cells.

Previous studies have revealed that ULK1 is ubiquitylated and degraded under nutrient starvation conditions, while Atg13 is ubiquitylated and degraded under normal nutrient conditions [16, 17]. We wondered whether the LPS-induced decrease in Atg13 in HSCs was dependent on UPS or on autophagy. We found that MG132 successfully increased the expression of Atg13 and inhibited the LPS-induced decrease in Atg13, while CHX decreased the expression of Atg13 and promoted the LPS-induced decrease of Atg13. Moreover, our data revealed that MG132 inhibited and CHX promoted LPS-induced inflammatory responses in LX2 cells. Treatment with SB203580 or MG132 reversed LPS-induced depletion of Atg13, and Anisomycin promoted the depletion of Atg13. Taken together, these data suggest that LPS promotes proteasome-dependent Atg13 degradation via p38 MAPK.

Considering the lack of data from experimental models, we collected liver tissues and serum from ACLF patients and performed the present study at cytology and molecular levels. In conclusion, the present study demonstrated the important role of Atg13 in regulating the inflammatory response in LX2 cells. Specifically, LPS leads to UPS-depletion of Atg13 via p38 MAPK in promoting inflammation. Moreover, the presence of Atg13 is of importance in maintaining the stability of ULK1 complex, which plays an essential role in the initiation of autophagy. These results provide a new Atg13-mediated link between autophagy and UPS. Regulation of Atg13 degradation has the potential to affect both the expanding repertoire of autophagy-dependent functions and the clinical efficacy of proteasome inhibitors utilized in inflammation treatments.

## Figures and Tables

**Table 1:** Demographic data and clinical characteristics of cirrhotic controls and ACLF patients.

Parameter	Cirrhotic controls (*n* = 30)	ALF (*n* = 30)	ACLF (*n* = 56)
Age (yr)	30.40 ± 6.96	37.23 ± 5.32	38.58 ± 5.19
Gender (M/F)	20/10	24/6	50/6
PTA (%)	85.29 ± 12.58	45.12 ± 11.27	32.26 ± 11.97
FIB (g/L)	3.04 ± 0.52	2.65 ± 0.51	2.70 ± 8.66
INR	1.07 ± 0.07	2.12 ± 0.34	2.46 ± 0.98
WBC (1 × 10^9^/L)	5.78 ± 1.31	7.56 ± 3.45	7.08 ± 3.50
PLT (1 × 10^9^/L)	229.40 ± 37.80	130.21 ± 21.35	95.82 ± 52.25
ALT (U/L)	12.33 ± 4.89	555.75 ± 501.25	620.38 ± 835.14
GLU (mM)	4.07 ± 0.29	6.23 ± 2.56	6.57 ± 3.13
TBIL (*μ*M)	8.17 ± 2.47	299.32 ± 178.23	304.09 ± 137.94
CHOL (mM)	3.58 ± 0.73	2.78 ± 0.56	2.25 ± 0.75
CREA (*μ*M)	50.47 ± 10.73	65.23 ± 16.56	78.91 ± 55.31
Na (mM)	140.80 ± 2.24	134.45 ± 13.57	135.32 ± 14.87
MELD		24.05 ± 4.89	23.82 ± 6.71

PTA: prothrombin activity; FIB: fibrinogen; INR: international normalized ratio; WBC: white blood cell count; PLT: platelet count; ALT: alanine aminotransferase; GLU: glucose; TBIL: total bilirubin; CHOL: cholesterol; CREA: creatinine; Na: sodium ions.

**Table 2:** Primers used in qRT-PCR.

Gene	Primers
Atg13	
R	5′-ATCGGCTAGCTAGCTACGATA-3′
F	5′-TAGGCGGCATGCGAGGCTACGC-3′
*β*-Actin	
F	5′-CCACACCCGCCACCAGTTCG-3′
R	5′-TACAGCCCGGGGAGCATCGT-3′

## Data Availability

The datasets used and/or analyzed during the current study are available from the corresponding author on reasonable request.

## Abbreviations

ACLF: Acute-on-chronic liver failure
HSCs: Hepatic stellate cells
UPS: Ubiquitin (Ub)-proteasome system
qRT-PCR: Quantitative real-time polymerase chain reaction
LPS: Lipopolysaccharide
SECs: Endothelial cells
ALS: Autophagy-lysosome system
Baf A1: Bafilomycin A1
CHX: Cycloheximide
ATGs: Autophagy-related proteins
MAPK: Mitogen-activated protein kinase
TLR4: Toll-like receptor 4.
